# Fine epitope mapping of glycoprotein Gn in Guertu virus

**DOI:** 10.1371/journal.pone.0223978

**Published:** 2019-10-16

**Authors:** Jingyuan Zhang, Abulimiti Moming, Xihong Yue, Shu Shen, Dongliang Liu, Wan-xiang Xu, Chen Wang, Juntao Ding, Yijie Li, Fei Deng, Yujiang Zhang, Surong Sun

**Affiliations:** 1 Xinjiang Key Laboratory of Biological Resources and Genetic Engineering, College of Life Science and Technology, Xinjiang University, Urumqi,China; 2 Center for Disease Control and Prevention of Xinjiang Uygur Autonomous Region, Urumqi, China; 3 State Key Laboratory of Virology, Wuhan Institute of Virology, Chinese Academy of Sciences, Wuhan, China; 4 NHC Key Lab. of Reproduction Regulation (Shanghai Institute of Planned Parenthood Research), Fudan University, Shanghai, China; University of Maryland School of Medicine, UNITED STATES

## Abstract

Guertu virus (GTV) is a tick-borne phleboviruses (TBPVs) which belongs to the genus *Banyangvirus* in the family of *Phenuiviridae*. In vitro and in vivo studies of GTV demonstrated that it was able to infect animal and human cell lines and could cause pathological lesions in mice. Glycoproteins (GP, including Gn and Gc) on the surface of Guertu virus (GTV) could bind to receptors on host cells and induce protective immunity in the host, but knowledge is now lacking on the information of B cell epitopes (BCEs) present on GTV-GP protein. The aim of this study was to identify all BCEs on Gn of the GTV DXM strain using rabbit pAbs against GTV-Gn. Seven fine BCEs and two antigenic peptides (APs) from nine reactive 16mer-peptides were identified, which are E_Gn_1 (^2^PIICEGLTHS^11^), E_Gn_2 (^135^CSQDSGT^141^), E_Gn_3 (^165^IP EDVF^170^), E_Gn_4 (^169^VFQEL K^174^), E_Gn_5 (^187^IDGILFN^193^), E_Gn_6 (^223^QTKWIQ^228^), E_Gn_7 (^237^CHKDGIGPC^245^), AP-8 (^299^GVRVRPKCYGFSRMMA^314^) and AP-9 (^355^CASH FCSSAESGKKNT^370^), of which six of mapped BCEs were recognized by the IgG-positive sheep serum obtained from sheep GTV-infected naturally. Multiple sequence alignments (MSA) based on each mapped BCE motif identified that the most of identified BCEs and APs are highly conserved among 10 SFTSV strains from different countries and lineages that share relatively close evolutionary relationships with GTV. The fine epitope mapping of the GTV-Gn would provide basic data with which to explore the GTV-Gn antigen structure and pathogenic mechanisms, and it could lay the foundation for the design and development of a GTV multi-epitope peptide vaccine and detection antigen.

## Introduction

Emerging pathogenic tick-borne viruses (TBVs) that can infect animals and humans have attracted much attention because of the increasing incidence of tick-borne viral diseases (TBVDs) and their significant impact on human health [1–3]. In recent years, two novel tick-borne phleboviruses (TBPVs), severe fever with thrombocytopenia syndrome virus (SFTSV), and Heartland virus (HRTV) have been shown to be correlated with severe human disease, having caused fatalities in East Asian countries and in the United States [4–7]. With the rapid development of metagenomics, more and more novel arboviruses have been identified from various hosts [8]. In 2014, Shen et al. [9] isolated one viral strain that displayed a strong evolutionary relationship with SFTSV from *Dermacentor nuttalli* ticks in the Xinjiang uygur autonomous region of China. Analysis of the phylogenetic tree constructed for this virus indicates that it belongs to the genus *Banyangvirus* in the family of *Phenuiviridae*, and named Guertu virus (GTV) [9, 10].

Wu et al. found the crystal structure of the envelope GP N-terminal (Gn) head domain from SFTSV and RVFV was compact and triangle-like, and the three subdomains comprising the Gn head domain have different arrangements [11]. The Gn protein plays a critical role in virion formation and in adhesion to new target cells [12], and helps viruses enter their target cells, which is the main target of neutralizing antibodies [13]. Thus, elucidating the B-cell epitopes (BCE) in the conserved domain or in the immunodominant region of GTV-Gn is very important to determine how the virus interacts with its host cells and to develop viral detection methods and vaccines [14].

The GTV genome contains three single-stranded minus-strand RNA fragments, comprising small (S), medium (M) and large (L) RNA fragments [9, 15]. As occurs with known *Banyangvirus* genus members, GP protein encoded by GTV-M gene is cut into two mature Gn and Gc proteins. However, knowledge about the antigenicity and immunodominant region of the GTV-encoded protein is still lacking.

Linear epitope mapping is an active area in viral investigation due to possible applications related to peptide vaccine development, antibody production, disease diagnosis and therapy [16]. So far there has been many epitope mapping approaches, such as recombinant (r-) DNA technology, chemically synthetic peptides or peptide chip method, and phage display libraries and so on [17–20]. However, a common defect of those methods is no way to identify an epitope minimal motif employing polyclonal antibodies (pAbs), and thus it is impossible to know entire BCEs on a target protein. Recently, the obstacle in epitope mapping has been overcome as the novel biosynthetic peptide (BSP) method with many merits was developed, in which short peptides (minimum 3mer peptide) fused with the truncated GST188 protein can be arbitrarily biosynthesized using the expression plasmid pXXGST-1 [21], Then the BSP method was further modified, which makes it more convenient to screen r- clones of encoding short peptides by SDS-PAGE analysis, that is, the screening of r-clones no longer requires to use control GST188 protein expressed by pXXGST-2 plasmid, when using newly constructed pXXGST-3 plasmid to express short peptides fused with GST188 carrier [22]. Xu et al. have used their own developed method to identify many fine BCEs on human zona pellucida glycoprotein-3 (hZP3) and human hZP4 using rabbit pAbs [21, 23], and especially to reveal three fine rabbit IgG-epitomes of human papilloma virus type 58 (HPV58) E6, E7 and L1 proteins for the first time [24]. In our previous studies, a lot of fine BCEs on the Crimean-Congo hemorrhagic fever virus (CCHFV) nucleoprotein (NP) and Gn were identified also using the method [25, 26], of which all the identified BCEs were recognized by anti-CCHFV positive sheep serum, confirming that the BSP method is credible and easy to operate for epitope mapping.

In this study, our main objectives are: 1) to reveal entire fine BCEs on Gn using rabbit pAbs against GTV-Gn; 2) to compare the similarity of immune responses to Gn between rabbit and sheep using positive serum from sheep GTV-infected naturally; 3) to determine the conservation of each mapped epitope among known SFTSV strains that share relatively close evolutionary relationships with GTV through multiple sequence alignment based on each fine BCE motif. Getting these results would provide the foundation for their possible applications related with developments of strain-specific diagnostic reagents and broad-spectrum multi-epitope peptide vaccine.

## Materials and methods

### Ethics statement

The study was approved by the Committee on the Ethics of Animal Experiments of Xinjiang Key Laboratory of Biological Resources and Genetic Engineering (BRGE-AE001), Xinjiang University. The animal serum samples were collected using random sampling and this process did not involving killing the animals.

### Plasmids, GTV strain and antibodies

The r-plasmid of pET-32a-Gn (aa 1–431) was previously constructed and stored by our research group. The prokaryotic expression plasmids of pXXGST-2 expressing GST188 protein and pXXGST-3 expressing short peptide fusion protein were kindly provided by Professor Wan-xiang Xu from Shanghai Institute of Planned Parenthood Research; The GTV in this study is based on GTV strain DXM (GenBank accession number: KT328592). Rabbit pAbs against GTV-Gn was kindly donated by Professor Fei Deng from Wuhan Institute of Virology, Chinese Academy of Sciences. Three New Zealand rabbits were injected intramuscularly with 0.5 mg of purified r-Gn protein and immunized at two-week intervals according to the conventional animal immune method. After the third immunization, rabbit antisera were collected and stored at -80°C until use [26]. The serum samples from sheep with or without infected-GTV were previously identified by indirect immunofluorescence assay (IFA) and reverse transcriptase polymerase chain reaction (RT-PCR) according to the references [9, 26]. Serum of a healthy sheep was used as negative controls in the Western blot assay. *Escherichia coli* BL21 (DE3) cells were used to express 16/8mer peptides fused with a truncated GST188 protein (i.e., with the initial 188 aa of GST) [21]. Goat anti-rabbit and mouse anti-goat IgG conjugated to horseradish peroxidase (HRP) were purchased from Beijing TransGen Biotech, Co., Ltd. (China).

### Other reagents and materials

DNA ligase and restriction enzymes *Bam*H I and *Sal* I (Takara Co., Ltd, Dalian, China), QIA quick Gel Extraction Kit (QIAGEN, Duesseldorf, Germany), unstained or pre-stained molecular weight markers (Thermo Fisher Science, Waltham, MA, USA), 0.2 μm nitrocellulose membrane (Whatman GmbH, Dossel, Germany), and enhanced chemiluminescence (ECL) plus western blotting detection kit (GE Healthcare, Buckinghamshire, UK) were obtained.

### Biosynthetic short peptides

**Fig 1:**
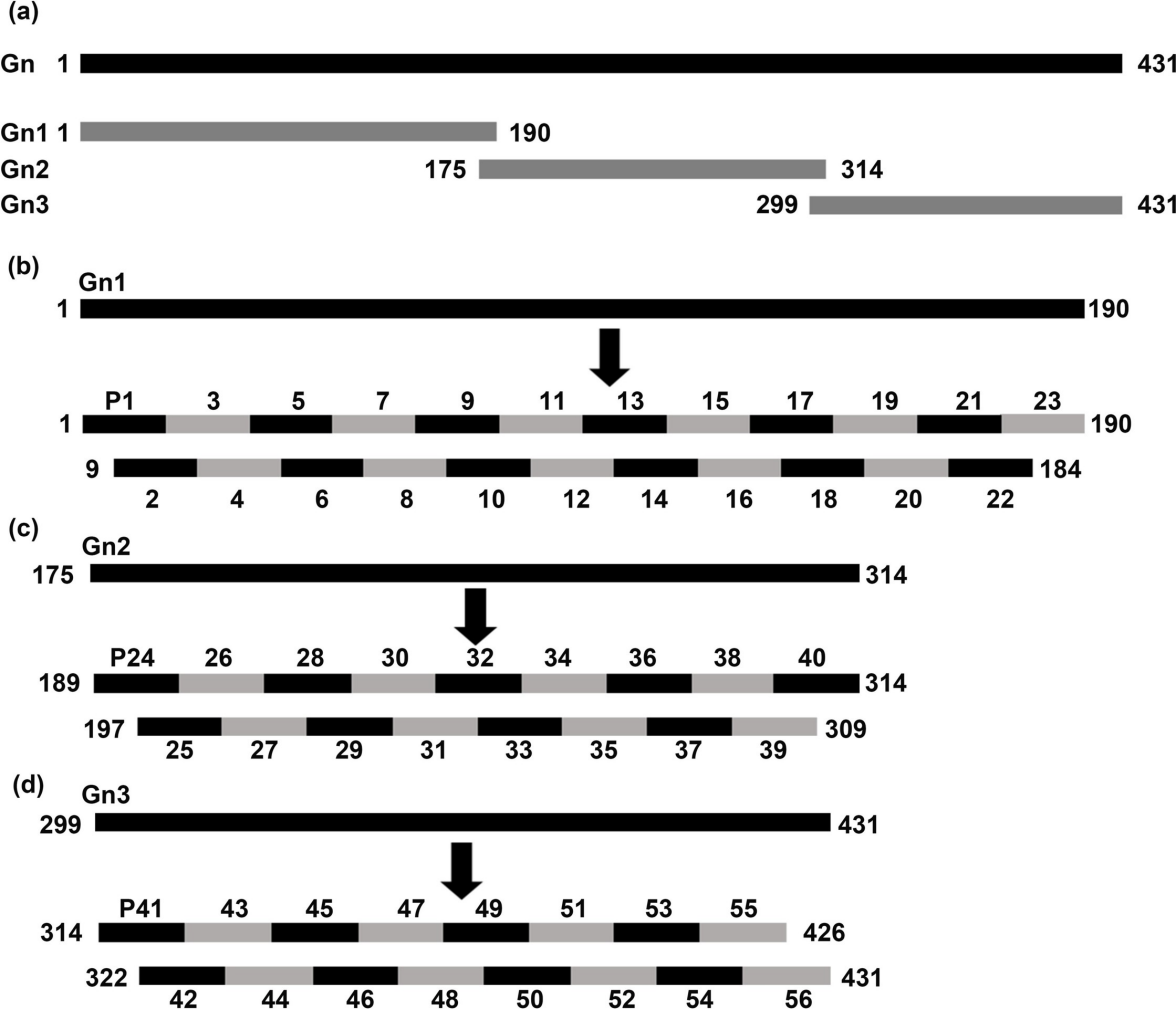
Schematic of epitope mapping strategy. (a) The black band indicates the full- length sequence of GTV-Gn, and the gray bands indicate three of truncated Gn1, Gn2 and Gn3 segments. Schematic of epitope mapping strategy involves 56 overlapping 16mer-peptides spanning Gn1 (b), Gn2 (c) and Gn3 sequences (d).

To conduct epitope mapping of Gn from GTV strain DXM (GenBank accession number: ALQ33264.1) using a modified BSP method, we used the feasible strategy shown in Fig 1, that is, the Gn with 431 amino acid (aa) residues was first truncated into fifty-six of overlapping 16mer-peptides numbering P1-P56 that all sequences were shown in S1 Table. Then the reactive 16mer-peptides shown in Western blotting will be further shortened into several sets of 8mer peptides with an overlap of 7 aa each other, as well as the overlapping longer 9-14mer peptides were designed for the reactive 16mer-peptides failed to find any reactive band of 8mer-peptides. The aa sequences of expressed 16/8mer peptides and their positions on Gn were shown in S1 and S2 Tables.

The DNA fragments encoding these short peptides were synthesized by Wuhan Tianyi Huiyuan Biotechnology Co., Ltd. Each fragment contained a *Bam*H I site at the 5' end, a *Sal* I cleavage site linked with a TAA termination codon at the 3' end. All DNA fragments encoding each short peptide were cloned into the pXXGST-3 vector expressing GST188-tagged protein in *E*. *coli*, respectively [21, 22].

### Expression of designed short peptides

The constructed plasmids expressing the overlapping peptides were transformed into *E*. *coli* BL21 (DE3) competent cells. Each r-clone was incubated in 3 mL LB medium containing 50 μg/mL ampicillin at 37°C and 220 rpm/min overnight. The next day, 30 μL of overnight bacteria culture was placed into 3 mL of fresh LB medium, grown at 30°C for 3.5 h to increase the bacterial density until reaching an optical density at 600 nm (OD_600_) of 0.6–0.7, and heat-induced to express each short peptide fusion protein at 42°C. To screen r-clones, the collected cell total proteins from each induced clone were analyzed by SDS-PAGE, and then those confirmed r-clones were sent to Ikang Biosciences Co., Ltd. for DNA sequencing. Finally, each collected cell pellets containing target peptide fusion protein was stored at -20°C, respectively.

### SDS-PAGE and Western blot analysis

After an r-clone was induced, its supernatant was removed by centrifugation in 2 mL of bacterial culture medium, and the cells were re-suspended by adding 160 μL of 1×PBS. Then 40 μL of 5×loading buffer was added and immersed in a water bath at 95°C for 10 min. Proteins were resolved by 12% SDS-PAGE gel electrophoresis. The gel was stained with Coomassie brilliant blue R-250 to analyze whether there is obvious band corresponding short peptide fusion protein, and then cell proteins were electrotransferred onto 0.2 μM nitrocellulose (NC) membrane for Western blotting. Regarding the specific antigen-antibody reaction, the NC membrane was blocked with 5% (w/v) skimmed milk powder in Tris-buffered saline-Tween 20 (TBS-T), incubated with rabbit anti-Gn pAbs (1:2500 dilution) or sheep sera (1:100 dilution) as the primary antibody overnight at 4°C, and then reacted with goat anti-rabbit IgG or mouse anti-goat IgG conjugated to horseradish peroxidase (HRP) at 1:2000 dilution as the secondary antibody. Finally, the NC membrane was washed with TBS-T and the blotted bands on it were developed by using the enhanced chemiluminescence (ECL) color reagent. A LAS-4000 hypersensitive chemiluminescence imager (Japan) was used to visualize immunoblots [22].

### Sequence analysis and 3D modeling

To analyze similarity of each mapped BCEs among homologous proteins, the Gn sequences of SFTSV strains from different countries and genetic lineages were downloaded from GenBank based on the phylogenetic tree of SFTSV strains [27].

The location of experimentally identified BCEs in the three-dimensional structure (3D) of the Gn protein was analyzed by PyMOL™ software (https://pymol.org/2/). The prediction of secondary structure was based on Garnier and Robson [28] as well as Chou and Fasman [29]. The hydrophilic scheme, flexible regimen, surface accessibility regimen and antigenicity index were analyzed and predicted using the methods of Kyte-Doolittle [30], Karplus-Schulz [31], Emini [32] and Jameson-Wolf [33].

## Results

### Mapping of antigenic peptides in GTV-Gn protein

**Fig 2:**
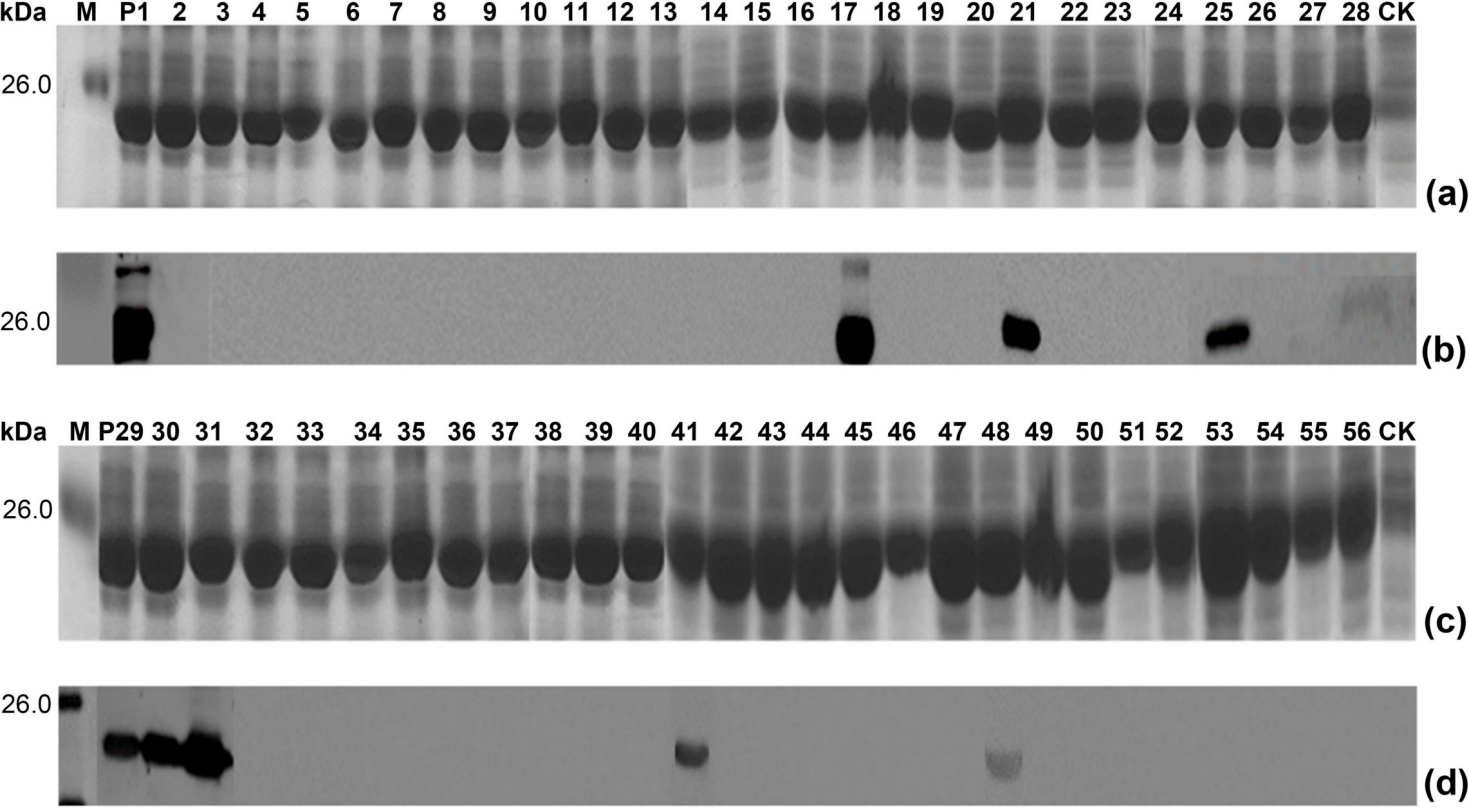
SDS-PAGE and Western blot analysis of expressed 16mer-peptides. (a, c) SDS-PAGE analysis of expressed 16mer-peptides. The numbers of P1-P56 indicate each 16mer-peptide in cell total proteins. The cell proteins of each r-clone were resolved by 12% SDS-PAGE gel electrophoresis and stained with Coomassie brilliant blue. M, the protein molecular marker; CK, Negative control of GST188 carrier protein expressed by pXXGST-2. (b, d) Western blot analysis for mapping reactive 16mer-peptides in P1-P56. The rabbit antiserum (1:2500 dilution) against GTV-Gn was used in Western blotting. The reactive bands in Western blotting were visualized by enhanced chemiluminescence.

Like strategy of chemically synthetic peptide method often used in epitope mapping, we first employed the pXXGST-3 expression plasmid to construct fifty-six of overlapping 16mer-peptides fused with the truncated GST188 carrier. DNA sequencing results showed that all biosynthesized 16mer-peptides with an overlap of 8 aa residues and covering the full-length sequence of GTV-Gn were correctly expressed. In the first round of antigenic peptide mapping, Western blotting results indicated that total nine 16mer-peptides of P1, P17, P21, P25 P29, P30, P31, P41 and P48 could specifically react to rabbit pAbs against GTV-Gn (Fig 2).

### Mapping of epitope motif in each antigenic peptides

**Fig 3:**
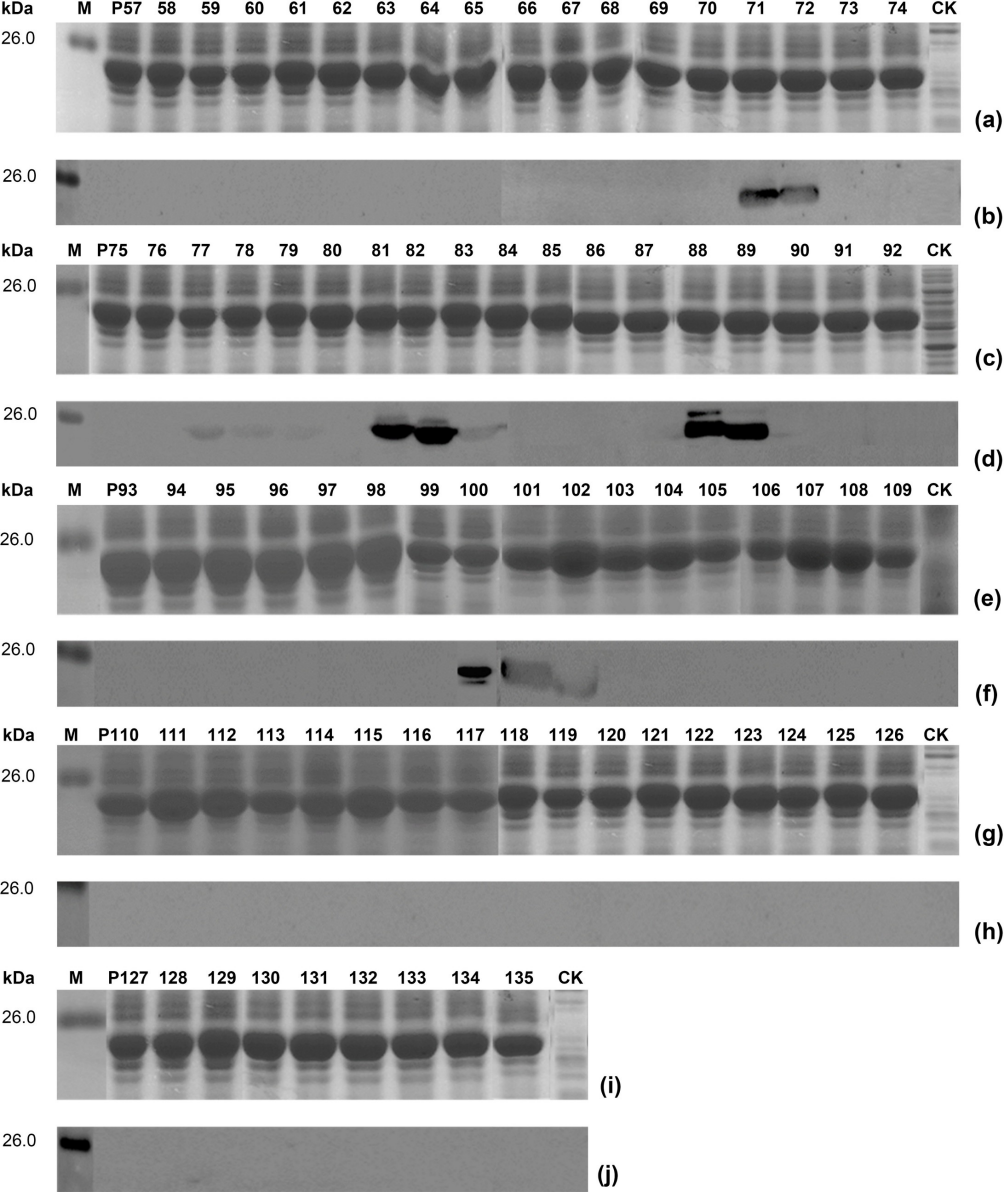
SDS-PAGE and Western blot analysis of expressed 8mer-peptides. (a, c, e, g, i) SDS-PAGE analysis of expressed 8mer-peptides. It indicates each 8mer-peptide for numbers P57-P65 of P1, P68-P74 of P17, P75-P83 of 21, P84-P92 of P25, P93-P109 of P29 and P30, P110-P117 of P31, P118-P126 of P41 and P127-P135 of P48. The cell proteins of each r-clone were resolved by 12% SDS-PAGE gel electrophoresis and stained with Coomassie brilliant blue. M, the protein molecular marker; CK, Negative control of GST188 protein. CP, Positive control of mapped reactive P1. (b, d, f, h, j) Western blot analysis for mapping fine epitopes in each reactive 16mer-peptides. The rabbit antiserum against GTV-Gn (1:2500 dilution) was used in Western blotting. The reactive bands in Western blotting were visualized by enhanced chemiluminescence.

It is imperative to identify an antibody-recognizing minimal motif in mapped antigenic peptides, because it is the essential foundation to delineate complete BCEs on a target protein. Therefore, it was constructed and expressed for nine sets of overlapping 8mer peptides after mapped reactive 16mer-peptides, which were used in the second round of fine epitope mapping. Several 8mer-peptides within them showed positive antigen-antibody reaction in Western blotting (Fig 3). Two 8mer-peptides of P71 and P72 in P17 were able to react specifically with rabbit pAbs, indicating that the minimum motif of named E_Gn_2 epitope is CSQDSGT because of the common sequence present in them. Similarly, of expressed overlapping 8mer-peptides from other eight reactive 16mer-peptides, P77-P79 and P81-P83 in P21, P88 and P89 in P25, and P100-P102 in P29 and P30 were founded to be able to react with rabbit pAbs as well (Fig 3). Obviously, this result indicated that there were four BCEs named E_Gn_3 (IPEDVF), E_Gn_4 (VFQELK), E_Gn_5 (IDGILFN) and E_Gn_6 (QTKWIQ), of which two were located in P21 due to existence of a non-reactive P80 between P77-P79 and P81-P83, one in P25 and one shared by P29 and P30, respectively.

### Mapping of epitope motif using longer 10/12mer-peptides

**Fig 4:**
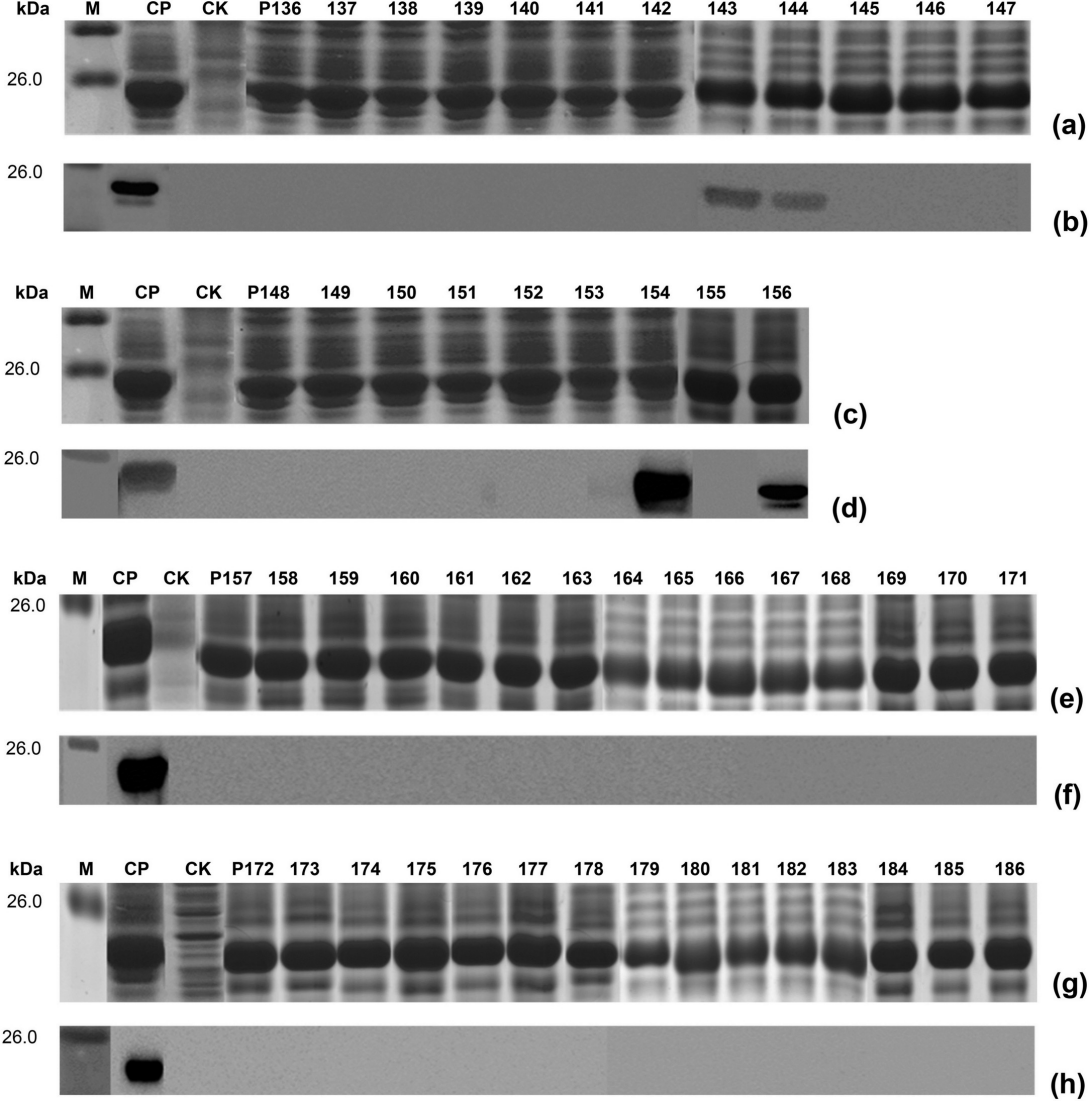
SDS-PAGE and Western blot analysis of expressed 9-14mer peptides. (a, c, e, g) SDS-PAGE analysis of expressed 9/10/12/14mer-peptides. It indicates each short peptide for numbers 10mer P136-P142 and 12mer P143-P147 of P1, 10mer P148-P154 and 9mer P155-P156 of P31, 10/12/14mer P157-P186 of P41 and P48. The cell proteins of each r-clone were resolved by 12% SDS-PAGE gel electrophoresis and stained with Coomassie brilliant blue. M, the protein molecular marker; CK, Negative control of GST188 carrier protein. CP, Positive control of P1. (b, d, f, h) Western blot analysis for mapping fine epitopes in each reactive 16mer-peptide. The rabbit antiserum against GTV-Gn (1:2500 dilution) was used in Western blotting. The reactive bands in Western blotting were visualized by enhanced chemiluminescence.

**Fig 5:**
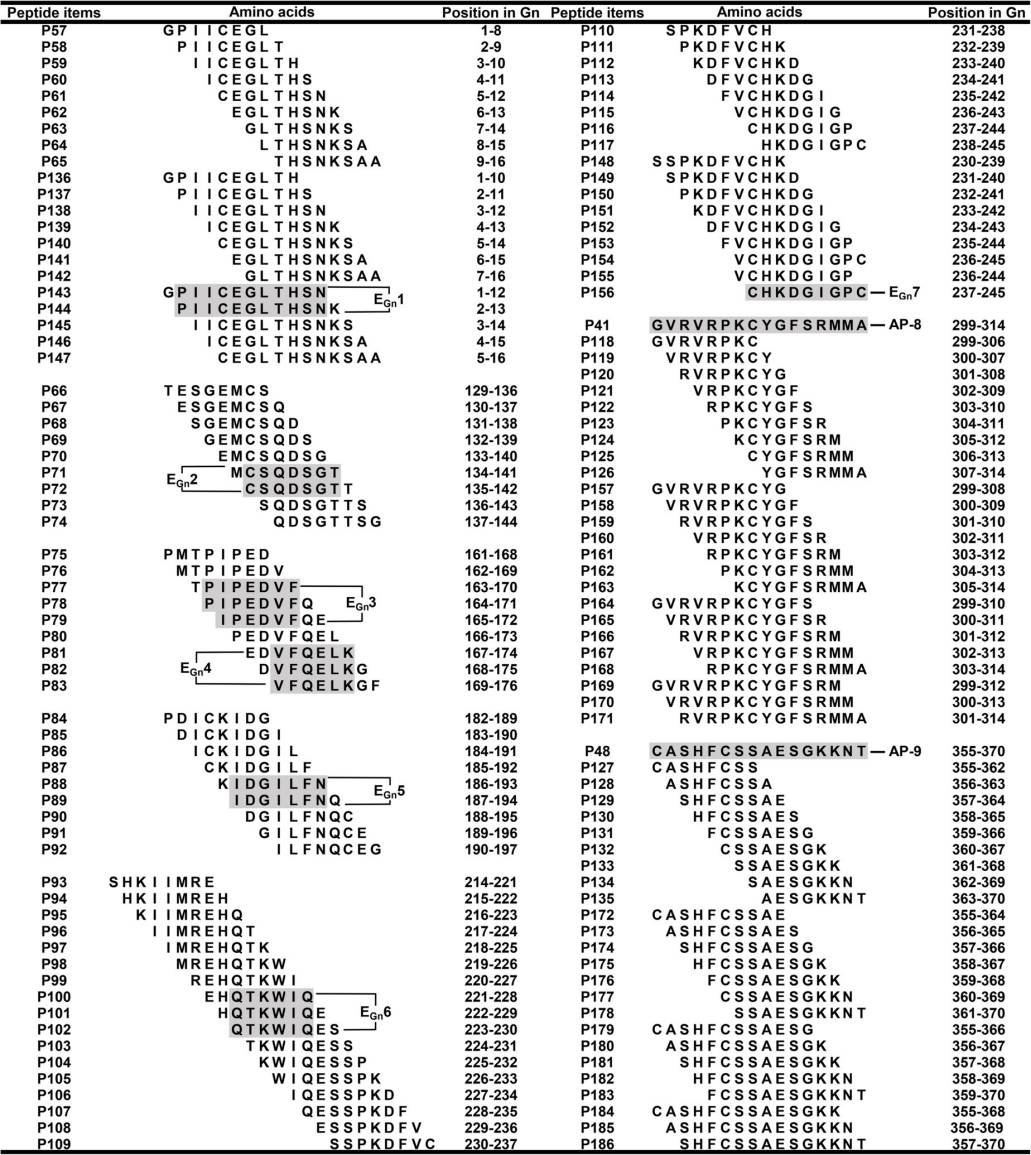
Determination of mapped epitope motif. Each mapped BCE minimal motif was determined according to the common sequence present in several overlapping peptides recognized by rabbit pAbs against Gn, which all reactive peptides were shown in shadows. (For interpretation of the references to color in this figure legend, the reader is referred to the web version of this article).

After did not define BCEs present in reactive P1, P31, P41 and P48 using four sets of 8mer-peptides of them, fifty one of longer overlapping short peptides were expressed for revealing their fine epitope in them, based on the known result that a precise nonapeptide epitope of HPV18-E6 protein was mapped by Western blotting (Xu WX, et al. Data unpublished). At first, the epitope named E_Gn_1 in P1 was identified using seven of 10mer-peptides (P136-P142) followed by five of 12mer-peptides (P143-P147), which was a BCE with 11 aa (PIICEGLTHSN) according to two reactive 12mer-peptides of P143 and P144. Secondly, another fine nonapeptide epitope (CHKDGIGPC) in P31 was mapped using seven of 10mer-peptides (P148-P154) followed by two of 9mer-peptides (P155, P156), which the latter was to be used for its BCE minimal motif identification. However, for two antigenic 16mer- peptides of P41 and P48, BCEs in them fail to be determined even using 10mer-, 12mer- and 14mer-peptides (P157-P186) as there was no any reactive band in them. It was shown in Figs 4 and 5 for the results of Western blot analysis using rabbit pAbs to Gn and fine BCE motif determination mentioned-above.

### Reactive profile of the mapped BCE with sheep antisera to GTV

**Fig 6:**
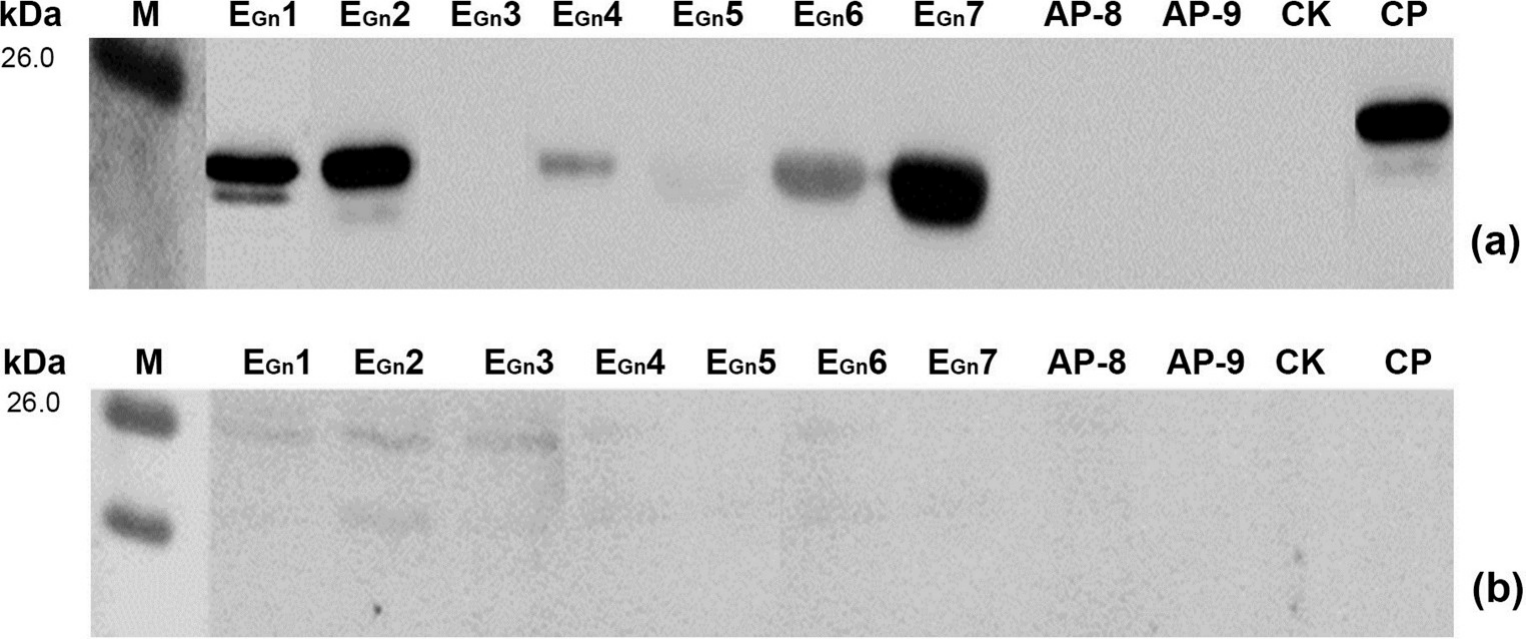
Western blot analysis of mapped BCEs and APs using sheep sera. (a) Using a positive serum from a sheep confirmed GTV-infection. (b) Using a serum from healthy sheep with no history of GTV infection as a negative control. CK, Negative control of GST188 protein. CP, Positive control of P1.

To determine whether the BCEs mapped by rabbit pAbs to Gn could also recognized by antiserum against GST from other host species, seven BCE peptides containing each BCE and two antigenic 16mer-peptides (AP-8 and AP-9) were utilized to perform Western blot analysis using the sera from sheep with or without GTV infection. Of them, six BCE peptides were able to react with positive serum from sheep in Western blotting (Fig 6A), while the serum of sheep without infected-GTV did not recognize nine reactive peptides of seven BCEs, AP-8 and AP-9 (Fig 6B). Of the nine peptides, four BCE peptides of E_Gn_1, E_Gn_2, E_Gn_6 and E_Gn_7 showed the strongest reaction with GTV-infected sheep serum and the reaction of E_Gn_4 and E_Gn_5 was weaker reaction in Western blotting (Fig 6A). Meanwhile, three peptides of E_Gn_3, AP-8 and AP-9 were not recognized by sheep positive serum (Fig 6A). In short, these results indicate that there is better similarity of the immune response to GTV-Gn between rabbit and sheep, and four BCE peptides of E_Gn_1, E_Gn_2, E_Gn_6 and E_Gn_7 can be used as candidate BCEs to develop the diagnostic antigen with single and/or multi-epitope peptide for sheep with GTV-infection.

### Sequence analysis and 3D localization of mapped epitopes

It is one of the main research objectives to determine conservation and specificity of each identified BCE among homologous proteins in epitope mapping. To achieve this, the multiple sequence alignment was conducted between the aa sequences of Gn^1-431^ from the GTV strain DXM and other 10 SFTSV strains from different countries and lineages. The SFTSV strains selected were representative of eight genetic lineages: C1 (China, JS4, ADZ04502.1), C2 (China, HNXY_130, AGI97068.1; China, HB 2014–33, ATG80983.1), C3 (China, HB155/China/2011, AFI45086.1), C4 (China, AHL/China/ 2011, AFJ15061.1), C5 (China, 42-Lu'an(CHN)-2015, AOO85596.1), J1 (Japan, SPL070A, BAQ59249.1; South Korean, KAGBH4, APT42341.1), J2 (KAC NH3, AJO16098.1) and J3 (China, ZJZHSH-HCY/China/06/2012, AMK05 827.1).

**Fig 7:**
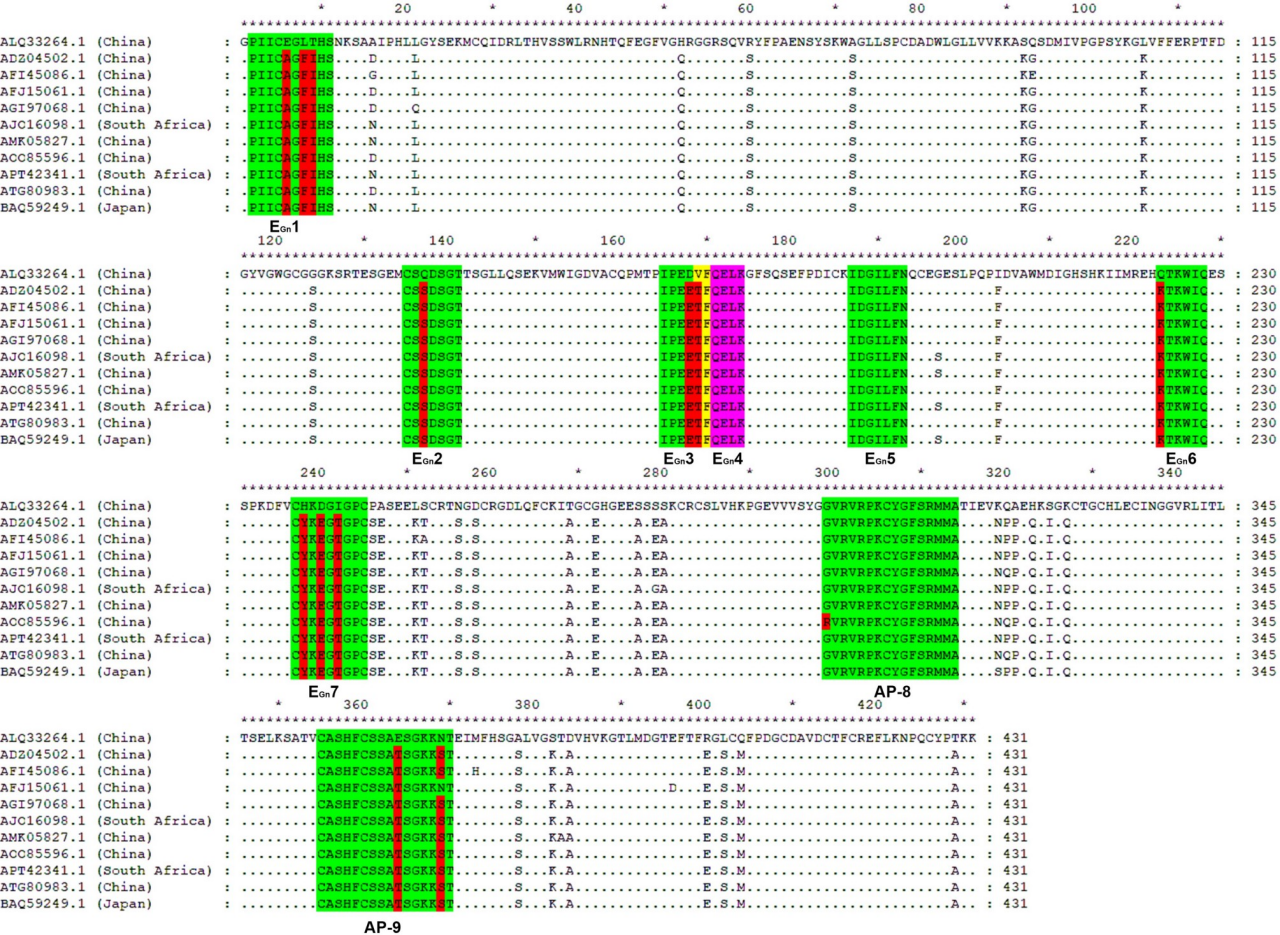
Sequence comparison between GST-Gn and 10 SFTSV strains. The GenBank codes and sources are shown at left and the sequence analysis was based on the ClustalW program. The nine of fine BCEs and APs recognized by pAbs are highlighted, and the variable aa residue within the BCE motif are highlighted in red. Dots (.) indicate identical aa residue in ten SFTSV strains (For interpretation of the references to color in this figure legend, the reader is referred to the web version of this article).

As shown in Fig 7, MSA analysis revealed that five BCE motifs were found to be of high sequence similarity among the 10 SFTSV strains (85.71% for E_Gn_2, 83.33% for E_Gn_4 and E_Gn_6, 93.75% for AP-8, and 87.50% for AP-9) except E_Gn_3, E_Gn_7 (66.67% sequence identity) and E_Gn_1 (70% sequence identity). The 8 BCE motifs have amino acid mutations, which are specifically shown in Fig 7. Moreover, the analysis results showed that the BCE E_Gn_5 and AP-8 were completely conserved within 10/9 SFTSV strains, indicating that they may be used as candidates for ‘universal’ GST and SFTSV multi-epitope vaccine design. The E_Gn_2 and E_Gn_6 BCEs were found to be highly conserved among homologous proteins (Fig 7), but the peptide (RTKWIC) similar to E_Gn_6 was not recognized by rabbit pAbs in Western blotting (S1 Fig), indicating that the E_Gn_6 is completely specific to the 10 SFTSV strains. For E_Gn_2, whether there is antibody cross-reactivity within homologous proteins remain to be determined using its similar peptide with one residue mutation. Interestingly, the E_Gn_1 and E_Gn_7 BCEs were specific for GST because of three residues mutation happened at the site corresponding to each BCE motif in homologous proteins of 10 SFTSV strains (Fig 7), indicating that they together with E_Gn_6 all can be used as diagnostic reagents to test GTV-infection.

**Fig 8:**
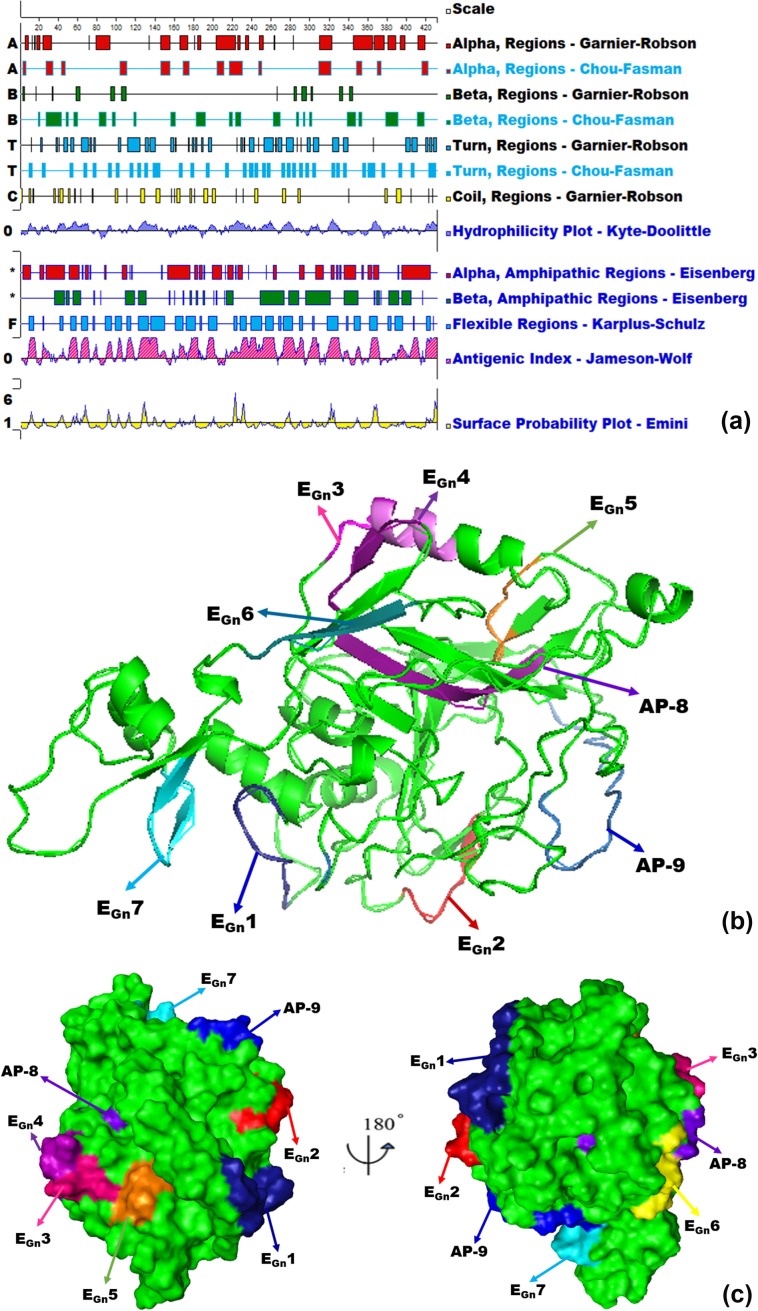
Prediction of Gn secondary structure and 3D localization of each BCEs. (a) Epitope prediction for GTV-Gn using DNAStar-Protean software. The secondary structure, flexibility plot, hydrophilicity, surface probability, and antigenicity index for GTV-Gn were taken into consideration. (b) The cartoon diagram shows the overall secondary structure of GTV-Gn was shown in different colors. The motifs indicate that seven BCEs and two APs were located on a well helix-angle-helix structure. (c) Location distribution on 3D structure of mapped BCEs and APs on molecular surface were shown in different colors. E_Gn_1 (deep blue), E_Gn_2 (red), E_Gn_3 (magenta), E_Gn_4 (violet), E_Gn_5 (orange), E_Gn_6 (yellow), E_Gn_7 (cyan), AP-8 (deep purple), AP-9 (sky blue). The figures were generated using the PyMOL™ molecular graphics system (For interpretation of the references to colour in this figure legend, the reader is referred to the web version of this article).

In this study, PyMOL™ software was used to simulate the 3D structure of GST- Gn to locate all the mapped BCEs and APs. Each BCE or AP was labeled with different color, for which two overlapping residues between E_Gn_3 and E_Gn_4 was shown in yellow (Fig 7). This result showed that all BCEs and APs were located on the surface of Gn-3D structure (Fig 8B and 8C), indicating the antibody accessibility of them. The secondary structure of the Gn^1-431^ was predicted using various methods [29–32] (Fig 8A). The predicted results indicated that there is no transmembrane domain and signal peptide sequence in Gn^1-431^. 3D models and protein secondary structure predictions indicated that the predicted results were consistent with the mapped BCEs and APs, and they were all located in the hydrophilic region (Fig 8A). In addition, based on the results obtained from these analyses, seven BCEs and two APs showed the features of good hydrophilicity, antibody accessibility, high flexibility and strong antigenicity, which would lay the foundation for selecting them as candidates for development of sheep GTV peptide vaccine or diagnostic antigen in the future.

## Discussion

Viral glycoproteins are involved in infection of the tick host, cytotropism in vertebrate hosts, high pathogenicity during the infection period in humans, and induce the generation of neutralizing antibody [34]. In the study on Hantaan virus, most of the neutralizing monoclonal antibodies (mAbs) were found to specifically target Gn and Gc proteins [35]. Compared to the glycosylation sites (^12^NKSA^15^ and ^42^NHSQ^45^) of the Gn from SFTSV HB29 strain, analysis of glycosylation sites (http://www.cbs.dtu.dk/services/NetNGlyc) of GTV-Gn in this study showed two N-glycosylation sites (^12^NKSA^15^ and ^42^NHTQ^45^). Cysteines responsible for disulfide bonds formation and multimerization, and two hydrophobic loops important for fusion are fully conserved among GP proteins of the GTV, SFTSV, and HRTV [9]. The key residue responsible for hydrogen bonds and salt bridges with an antibody is conserved in GTV- and SFTSV-Gn proteins, suggesting that GTV-Gn is likely to be recognized by the antibody derived from SFTSV [9]. However, there are still no reports on the antigenic region of the GTV-Gn. To investigate antigenic sites on GTV-Gn, epitope mapping of the Gn was performed by using rabbit pAbs and the improve BSP method [22], by which seven fine BCEs and two APs were identified. Interestingly, the E_Gn_1 motif is found to be closely linked to the first glycosylation site (^12^NKSA^15^) of GST-Gn (Fig 7), suggesting that the antibody induced by E_Gn_1 might prevent glycosylation of this site through the inhibition of spatial conformation, and the biological/immunological significance of the mapped BCE remains to be studied in the future.

It has been one of important objectives in the study of epitope mapping to understand the similarity and specificity of immune response to a target antigen between different species for future possible application of mapped BCEs usually using rabbit pAbs. Thus, the antiserum of sheep GTV-infection was utilized to compare the Gn antigenicity between rabbit and sheep in this work. As shown in Fig 6, 6 of mapped 9 BCEs could reacted with sheep antiserum to GTV, and three (E_Gn_3, AP-8 and AP-9) did not show immunogenic in sheep, which the results clearly reflected the better similarity and some differences of the immune response to GTV-Gn in rabbit and sheep. It is a common phenomenon for many species studied; for instance, the sheep serum to peste des petits ruminants virus (PPRV) could recognize 9 of 13 BCEs on hemagglutinin (H) protein of PPRV [36], and the murine pAbs to HPV58 L1-VLP could react with 13 of 18 BCEs mapped by rabbit pAbs to r-HPV58-L1 [24]. Notably, the completely similar antibody-responses to a given antigen also existed between various species sometimes; for instance, all rabbit IgG-BCEs on NPs of CCHFV/PPRV could react with the antisera from sheep CCHFV or PPRV-infected [25, 37], and the antibodies to three of β-hCG BCEs present in a chimeric peptide immunogen all were generated in both rabbit and mice [38]. These results indicate that the immune systems of rabbit, murine and sheep could recognize most and/or all of antigenic sites present in a given antigen despite differing greatly in biology and genetics for them, and thus can explain why there are too many reports on identifying BCEs on various viral proteins with rabbit and mouse antisera. To our knowledge, there are few reports about the immunogenic comparison of viral proteins between human and rabbit or murine as mentioned above, although many researches were carried employing the sera from patients or convalescents with various virus infection for epitope mapping [39–41]. In this work, we also tried to investigate whether a conserved E_Gn_5 could be recognized by the serum of a patient with infected-SFTSV (because of no sera from patient infected-GTV), but failed to achieve the desired result. Because it is an interesting scientific question and the need of future application to know similarity of mapped BCEs within rabbit and human, the study will be continued if we could collect the human antisera to GTV later on.

In the present paper, the results of multiple sequence alignment between the GTV strain and 10 SFTSV strains indicated that nine of BCEs and APs shared 66.67%-100% sequence identity with the homologous proteins from SFTSV (Fig 7), Considering that the hydrophilic region and antigenic region are more susceptible to exposed to the immune system, it could display higher reactivity when contacted with pAbs. Thus, we analyzed the hydrophilicity and antigenic region of those linear BCEs. Our results clearly indicated that all mapped BCEs are located in the hydrophilic domains and six of the nine identified BCEs from this study are consistent with the result predicted for the antigenic BCEs (Fig 8A). When these BCEs are located in the constructed 3D model, it is obvious that they occupy a relatively large and accessible surface area (Fig 8B and 8C), which is consistent with the expected. This suggests that the epitope prediction tool combined with the biosynthetic peptide method is reliable, which could reduce the work load and cost of epitope mapping in immune diagnosis and fine BCE motif recognition [26]. However, whether the identified BCEs could improve the sensitivity of virus detection and be efficacious in a vaccine remains to be further investigation.

Epitope mapping methods are based on recombinant DNA [17], protein display libraries [42], and other methods. The BSP method used in this study shows many advantages of easy operation, simple, cheap, reliable, and adaptable, of which another outstanding advantage is to help in discriminating reactive bands in Western blotting, as those expressed peptides were located in a weak antigenic area of cell total proteins, and thus avoid the false positive bands associated with two bacterial strong antigenic proteins with 23 kDa and 31 kDa [22]. The BSP method can be used under standard laboratory conditions, making it a useful for fine BCE motifs mapping with pAbs [37, 43]. For example, Yu et al. [37] identified 19 linear BCEs on H protein encoded by PPRV and Liu et al. [43] mapped 5 BCEs on NP2 from CCHFV that all were recognized by CCHFV-positive sheep serum, respectively. In addition, the longest linear BCE (E_Gn_1) with 11 residues was identified in Western blotting with the BSP method. The result in the present study indicates that six BCEs of E_Gn_1, E_Gn_2, E_Gn_4, E_Gn_5, E_Gn_6 and E_Gn_7 were specifically recognized by antibody-positive sera from sheep with GTV infection in Western blotting (Fig 6), but the E_Gn_3, AP-8 and AP-9 did not. It is suggested that the antigenic similarity and differences between both rabbit and sheep species.

Wu et al. [11] have studied the complicated structure of the viral head Gn envelope protein of SFTSV and the activities of neutralizing antibodies against it, and found that the Gn-containing head is similar to that of RVFV that it comprises a compact triangle with different arrangements (Subdomain Ⅰ, II and III). Subdomain III is recognized by specific neutralizing antibodies, and subdomain II is mainly recognized by broad-spectrum neutralizing antibodies [11]. Although SFTSV has a close evolutionary relationship with GTV, current investigations on the GTV-Gn protein structure are still not complete. We speculated that the corresponding domain of GTV-Gn protein has similar characteristics to that of SFTSV. MSA indicated that seven BCEs and two APs were presumed to be located in the subdomain II and III of the GTV-Gn, could be recognized by broad-spectrum neutralizing antibodies. However, whether the identified seven BCEs and two APs of GTV-Gn in this study have neutralizing activity still requires further verification.

In conclusion, seven fine BCEs and two 16mer-APs on Gn from GTV stain DXM were identified with rabbit pAbs against r-Gn, of which six of BCEs can be recognized by the antiserum from sheep infected-GTV, and four BCEs are complete or highly conserved among homologous proteins of selected representative SFTSV strains. These results improve our understanding of the antigenic characteristics of the GTV-Gn protein, and will also contribute to the development of GTV/SFTSV detection agents and preventive GTV r-peptide vaccines based on epitopes.

## Supporting information

S1 Table 16mer peptides amino acid sequence and their location on GTV strain DXM Gn. (DOC)

S2 Table 8mer peptides amino acid sequence and their location on GTV strain DXM Gn. (DOC)

S1 Fig Western blot analysis of mutated or shorten EGn6 using rabbit pAbs to GTV-Gn. Lane 1~6: _1_E_Gn_6 (^224^TKWIQ^228^); _2_E_Gn_6 (^225^KWIQ^228^); _3_E_Gn_6 (^226^WIQ^228^); _4_E_Gn_6 (^223^QTKWIQ^228^); _5_E_Gn_6 (^223^QTKWIQ^228^); _Δ_E_Gn_6 (^223^KTKWIQ^228^); CK: Negative control of expressed GST188 carrier protein; CP: positive control used E_Gn_6. M, Protein molecular marker; CK, Negative control of GST188 protein. CP, Positive control of CP1. The rabbit antiserum against GTV-Gn (1:2500 dilution) was used in Western blotting. The reactive bands in Western blotting were visualized by enhanced chemiluminescence.
Note: The sequence of _Δ_E_Gn_6 originates from the homologous proteins of SFTSV.
(TIF)
