# Supplementary material for: Fine epitope mapping of glycoprotein Gn in Guertu virus
Source: PLoS One. 2019 Oct 16;14(10):e0223978. doi: 10.1371/journal.pone.0223978 (PMC6795428; doi:10.1371/journal.pone.0223978)
Supplement: S1 Table — (DOC) [file pone.0223978.s001.doc]

**S1 Table.** **16mer peptides aa sequence and their location on GTV strain DXM Gn.**

| Peptide items | Position in Gn | Amino acids | Peptide items | Position in Gn | Amino acids |
| --- | --- | --- | --- | --- | --- |
| P1 | Gn^1-16^ | GPIICEGLTHSNKSAA | P29 | Gn^214-229^ | SHKIIMREHQTKWIQE |
| P2 | Gn^9-24^ | THSNKSAAIPHLLGYS | P30 | Gn^222-237^ | HQTKWIQESSPKDFVC |
| P3 | Gn^17-32^ | IPHLLGYSEKMCQIDR | P31 | Gn^230-245^ | SSPKDFVCHKDGIGPC |
| P4 | Gn^25-40^ | EKMCQIDRLIHVSSWL | P32 | Gn^238-253^ | HKDGIGPCPASEELSC |
| P5 | Gn ^33-48^ | LIHVSSWLRNHTQFEG | P33 | Gn^246-261^ | PASEELSCRTNGDCRG |
| P6 | Gn ^41-56^ | RNHTQFEGFVGHRGGR | P34 | Gn^254-269^ | RTNGDCRGDLQFCKIT |
| P7 | Gn^49-64^ | FVGHRGGRSQVRYFPA | P35 | Gn^262-279^ | DLQFCKITGCGHGEES |
| P8 | Gn^57-72^ | SQVRYFPAENSYSKWA | P36 | Gn^270-285^ | GCGHGEESSSSKCRCS |
| P9 | Gn^65-80^ | ENSYSKWAGLLSPCDA | P37 | Gn^278-293^ | SSSKCRCSLVHKPGEV |
| P10 | Gn^73-88^ | GLLSPCDADWLGLLVV | P38 | Gn^286-301^ | LVHKPGEVVVSYGGVR |
| P11 | Gn^81-96^ | DWLGLLVVKKASQSDM | P39 | Gn^294-309^ | VVSYGGVRVRPKCYGF |
| P12 | Gn^89-104^ | KKASQSDMIVPGPSYK | P40 | Gn^302-314^ | VRPKCYGFSRMMA |
| P13 | Gn^97-112^ | IVPGPSYKGLVFFERP | P41 | Gn^299-314^ | GVRVRPKCYGFSRMMA |
| P14 | Gn^105-120^ | GLVFFERPTFDGYVGW | P42 | Gn^307-322^ | YGFSRMMATIEVKQAE |
| P15 | Gn^113-128^ | TFDGYVGWGCGGGKSR | P43 | Gn^315-330^ | TIEVKQAEHKSGKCTG |
| P16 | Gn^121-136^ | GCGGGKSRTESGEMCS | P44 | Gn^323-338^ | HKSGKCTGCHLECING |
| P17 | Gn^129-144^ | TESGEMCSQDSGTTSG | P45 | Gn^331-346^ | CHLECINGGVRLITLT |
| P18 | Gn^137-152^ | QDSGTTSGLLQSEKVM | P46 | Gn^330-354^ | GVRLITLTSELKSATV |
| P19 | Gn^145-160^ | LLQSEKVMWIGDVACQ | P47 | Gn^347-362^ | SELKSATVCASHFCSS |
| P20 | Gn^153-168^ | WIGDVACQPMTPIPED | P48 | Gn^355-370^ | CASHFCSSAESGKKNT |
| P21 | Gn^161-176^ | PMTPIPEDVFQELKGF | P49 | Gn^363-378^ | AESGKKNTEIMFHSGA |
| P22 | Gn^169-184^ | VFQELKGFSQSEFPDI | P50 | Gn^371-386^ | EIMFHSGALVGSTDVH |
| P23 | Gn^177-190^ | SQSEFPDICKIDGI | P51 | Gn^379-394^ | LVGSTDVHVKGTLMDG |
| P24 | Gn^174-189^ | KGFSQSEFPDICKIDG | P52 | Gn^387-402^ | VKGTLMDGTEFTFRGL |
|  |  |  |  |  | **Continued S1 Table** |
| Peptide items | Position in Gn | Amino acids | Peptide items | Position in Gn | Amino acids |
| P25 | Gn^182-197^ | PDICKIDGILFNQCEG | P53 | Gn^395-410^ | TEFTFRGLCQFPDGCD |
| P26 | Gn^190-205^ | PDICKIDGILFNQCEG | P54 | Gn^403-418^ | CQFPDGCDAVDCTFCR |
| P27 | Gn^198-213^ | ESLPQPIDVAWMDIGH | P55 | Gn^411-426^ | AVDCTFCREFLKNPQC |
| P28 | Gn^206-221^ | VAWMDIGHSHKIIMRE | P56 | Gn^419-431^ | EFLKNPQCYPTKK |

Note: Peptide item P23 is 14mer peptide, P40 and P56 are 13mer peptide. All segments refer to the previous truncated segments design.
