# Supplementary material for: Fine epitope mapping of glycoprotein Gn in Guertu virus
Source: PLoS One. 2019 Oct 16;14(10):e0223978. doi: 10.1371/journal.pone.0223978 (PMC6795428; doi:10.1371/journal.pone.0223978)
Supplement: S2 Table — (DOC) [file pone.0223978.s002.doc]

**S2 Table. 8mer peptides aa sequence and their location on GTV strain DXM Gn.**

|  | 8mer peptides in each reactive peptide | | | | | | | |  |
| --- | --- | --- | --- | --- | --- | --- | --- | --- | --- |
|  | P1  (P57-P65) | P17  (P66-P74) | P21  (P75-P83) | P25  (P84-P92) | P29  (P93-P100) | P30  (P101-P109) | P31  (P110-P117) | P41  (P118-P126) | P48  (P127-135) |
|  | GPIICEGL | TESGEMCS | PMTPIPED | PDICKIDG | SHKIIMRE | HQTKWIQE | SPKDFVCH | GVRVRPKC | CASHFCSS |
|  | PIICEGLT | ESGEMCSQ | MTPIPEDV | DICKIDGI | HKIIMREH | QTKWIQES | PKDFVCHK | VRVRPKCY | ASHFCSSA |
|  | IICEGLTH | SGEMCSQD | TPIPEDVF | ICKIDGIL | KIIMREHQ | TKWIQESS | KDFVCHKD | RVRPKCYG | SHFCSSAE |
|  | ICEGLTHS | GEMCSQDS | PIPEDVFQ | CKIDGILF | IIMREHQT | KWIQESSP | DFVCHKDG | VRPKCYGF | HFCSSAES |
|  | CEGLTHSN | EMCSQDSG | IPEDVFQE | KIDGILFN | IMREHQTK | WIQESSPK | FVCHKDGI | RPKCYGFS | FCSSAESG |
|  | EGLTHSNK | MCSQDSGT | PEDVFQEL | IDGILFNQ | MREHQTKW | IQESSPKD | VCHKDGIG | PKCYGFSR | CSSAESGK |
|  | GLTHSNKS | CSQDSGTT | EDVFQELK | DGILFNQC | REHQTKWI | QESSPKDF | CHKDGIGP | KCYGFSRM | SSAESGKK |
|  | LTHSNKSA | SQDSGTTS | DVFQELKG | GILFNQCE | EHQTKWIQ | ESSPKDFV | HKDGIGPC | CYGFSRMM | SAESGKKN |
|  | THSNKSAA | QDSGTTSG | VFQELKGF | ILFNQCEG |  | SSPKDFVC |  | YGFSRMMA | AESGKKNT |
| BCE/AP | E_Gn_1 | E_Gn_2 | E_Gn_3 | E_Gn_4 | E_Gn_5 | E_Gn_6 | E_Gn_7 | AP-8 | AP-9 |
