# Supplementary figures and images for: Fine epitope mapping of glycoprotein Gn in Guertu virus
Source: PLoS One. 2019 Oct 16;14(10):e0223978. doi: 10.1371/journal.pone.0223978 (PMC6795428; doi:10.1371/journal.pone.0223978)

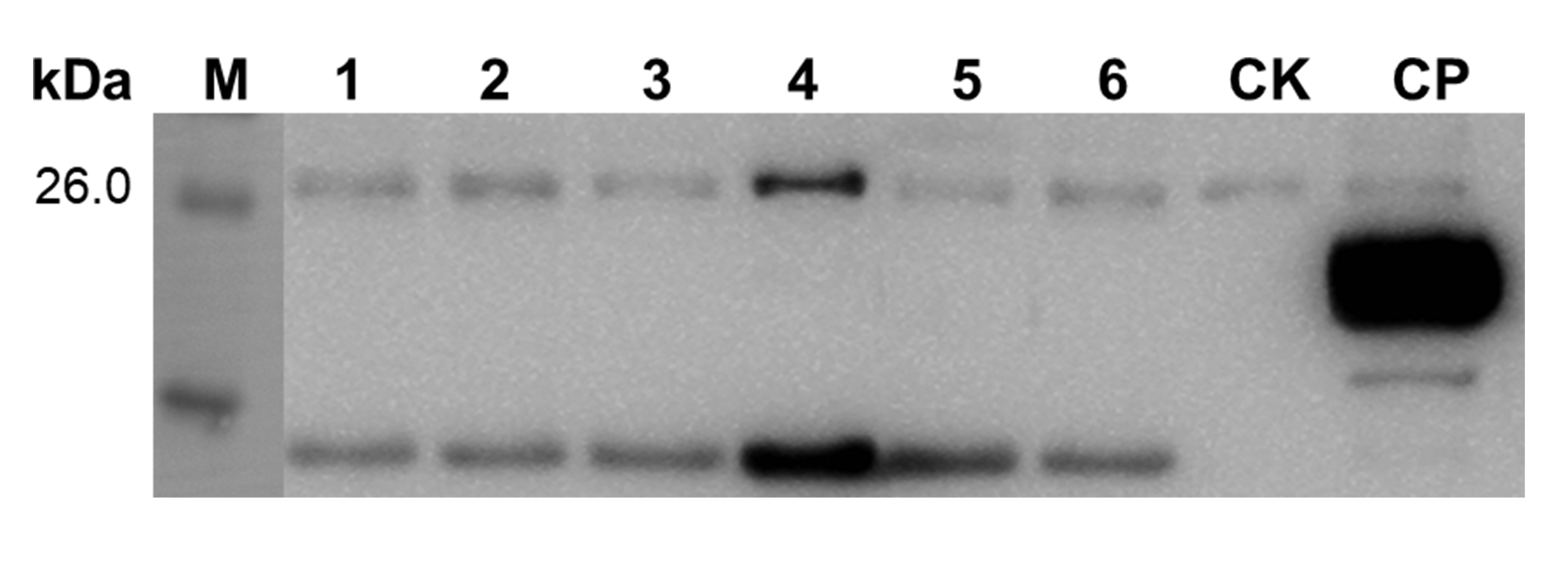

Supplement: S1 Fig — Lane 1~6: 1EGn6 (224TKWIQ228); 2EGn6 (225KWIQ228); 3EGn6 (226WIQ228); 4EGn6 (223QTKWIQ228); 5EGn6 (223QTKWIQ228); ΔEGn6 (223KTKWIQ228); CK: Negative control of expressed GST188 carrier protein; CP: positive control used EGn6. M, Protein molecular marker; CK, Negative control of GST188 protein. CP, Positive control of CP1. The rabbit antiserum against GTV-Gn (1:2500 dilution) was used in Western blotting. The reactive bands in Western blotting were visualized by enhanced chemiluminescence. Note: The sequence of ΔEGn6 originates from the homologous proteins of SFTSV. (TIF) [file pone.0223978.s003.tif]
